# Perception and satisfaction in patients using clear aligners versus fixed orthodontic appliances

**DOI:** 10.6026/973206300220770

**Published:** 2026-02-28

**Authors:** Prashant Patil, Girish Polavaram Vasudeva Setty, Bareera Shafiuddin, Altaf Hussain, Sunanda Paul, Riyas Karim6, Md. Kafeel Ahmed

**Affiliations:** 1 Department of Orthodontics, Al Badar Dental College and Hospital, Gulbarga, Karnataka, India; 2 Neuromuscular Orthodontist, Private Practice, Smile Architect Dental, Bangalore, Karnataka, India; 3 Orthodontist Consultant, Doha, Qatar; 4 Specialist Orthodontist, Qimat Altieb Medical Complex, Riyadh, Saudi Arabia; 5 Department of Orthodontics Dentofacial and Orthopaedics, Government Dental College and Hospital, Silchar, Assam, India; 6 Department of Periodontics, Indira Gandhi Institute of Dental Sciences, Kothamangalam, Kochi, Kerala, India; 7 Department of Periodontology and Implantology, MNR Dental College and Hospital, Sangareddy, Hyderabad, Telangana, India

**Keywords:** Clear aligners, fixed orthodontic appliances, patient satisfaction, aesthetic perception, oral health-related quality of life, adult orthodontics

## Abstract

Patient satisfaction drives orthodontic treatment success, yet clear aligners (CA) versus fixed appliances (FA) comparisons remains inconclusive. This prospective cohort study assessed psychosocial impact, satisfaction and OHRQoL in 120 patients over 12 months using validated questionnaires at multiple timepoints. CA users reported significantly higher aesthetic satisfaction throughout (p<0.001), with 78.3% rating aesthetics excellent vs 41.7% in FA at 12 months. Comfort scores strongly favored CA (p<0.001), while oral health- related quality of life (OHRQoL) was comparable at endpoint (p=0.342) despite similar treatment durations. Clear aligners advance orthodontic practice as an aesthetically superior, comfortable alternative that matches fixed appliance functional outcomes.

## Background:

There has been a paradigm shift in orthodontics treatment in the last twenty years where conventional fixed appliances have been replaced with less conspicuous options. The clear aligner therapies, which align technology introduced in the late 1990s, has grown exponentially to reach about 20-30 percent of the orthodontic adult market by 2023 [1]. The surge represents the growing patient demand in the treatment options that are less psychosocially and aesthetically damaging in the context of active therapy [2]. At the same time, traditional fixed appliances have been updated and now have ceramic brackets and specially made lingual systems, but still have visible metal parts, which do not appeal to adult patients pursuing treatment [3]. The patient-centered outcomes are designed as the key indicators of modern orthodontic studies due to the understanding that technical perfection should correspond to the subjective satisfaction to guarantee the success of the treatment [4]. The perception of a patient would affect his or her compliance, attendance at appointments, and eventually clinical outcomes. Various studies have examined parameters of satisfaction, and the results found that the parameters that adults value the most are aesthetic, comfort, and less lifestyle disruption instead of speed of treatment or cost [5]. Nevertheless, the current literature demonstrates contradictory results on the quality of clear aligner's superiority. Although there are studies which show better satisfaction with clear aligners as they can be removed and no one notices them [6]. There are studies which show similar or even lower satisfaction rates owing to slower progress and higher compliance rates [7]. Clear aligners have significant benefits in quality of life in the first stages of treatment but could not establish the benefits of long-term rewards [8]. On the other hand, previous studies have reported a vastly greater aesthetic satisfaction rating of clear aligners at 6 and 12 months, but the oral health-related quality of life (OHRQoL) disparities faded at the conclusion of therapy [9]. Such discrepancies are probably due to the heterogeneity in methods, such as different measurement tools, absence of standardized intervals of follow-up, and inability to address such confounding factors as the severity of malocclusion and the experience of the provider [10]. Moreover, the majority of comparative researches have based on retrospective designs or single-centre cohorts, which have brought in the selection bias and restricted generalizability. The interplay between the treatment modality and particular patient-reports domains- such as social impact and self-esteem and functional limit- is not well defined. Although aesthetic issues prevail in the short-term motivation, oral hygiene care, and perceived efficacy might have a greater role in the long-term satisfaction [11]. An in-depth assessment using reliable psychometric instruments over a span of time is fundamental in making evidence-based treatment suggestions. Therefore, it is of interest to compare the patient perception and satisfaction between clear aligners and fixed appliances through the validated questionnaires and visual analogue scales during 12 months treatment.

## Materials and Methods:

## Study design and setting:

This is a prospective, parallel-group cohort study that was carried out at the Department of Orthodontics, in March 2021- February 2024.

## Sample size calculation:

Sample size was calculated with reference to pilot study of 20 patients who found a mean difference of 15 points in the total satisfaction scores between the groups (SD 20). The minimum possible sample was 29 patients in each group using independent t-test and 0.05 as the 0.05, power=0.80 and effect size=0.75. The figure was 120 patients (60 each group) to take into consideration the 15% attrition.

## Participant selection:

Adult patients (18-45 years) who have been referred to the clinic consecutively because of seeking comprehensive orthodontic treatment were screened. The inclusion criteria included: Angle Class I or II malocclusion that needs non-extraction treatment; good periodontal health (pocket depth 3 mm or less, no bleeding on probing); intent to adhere to the study procedures; and taking of baseline psychometric measures. The exclusion criteria were: history of orthodontic therapy; caries (at least 3 lesions); severe skeletal discrepancies that need surgical correction; smoking; systemic disease which interferes with oral health; and pregnant state.

## Treatment allocation:

Selecting treatment modality was done self-selection by patients, having been fully counselled on costs, expectations of treatment and compliance requirements. Groups were then matched at age, gender, level of malocclusion (Peer Assessment Rating [PAR] scores) and level of baseline aesthetic concern to reduce selection bias. The procedures were carried out by two skilled orthodontists (more than 10 years' experience and at least 500 cases per modality) according to the standardized protocols.

## Intervention protocols:

## Clear Aligner Group:

The patients underwent an intervention of clear aligner therapy (Invisalign, Align Technology) with aligners changed every week. The placement of attachment was according to the recommendations of the manufacturer. The patients were asked to use the aligners 22 hours a day, and only take them off in the course of eating and oral care. The visits to the office were done at 6-week intervals. Fixed Appliance Group: The patients used traditional fixed preset edgewise appliances (0.022-inch slot, MBT prescription) with metal brackets. The first archwires were made of 0.014-inch nickel-titanium, followed by stainless steel. The adjustment meetings were to be done after every 4 weeks. They were all given oral hygiene training and regular dietary counselling.

## Outcome measures:

## The patient satisfaction and perception outcomes were measured using:

[1] Psychosocial Impact of Dental Aesthetics Questionnaire (PIDAQ): 23-item validated questionnaire of dental self-confidence, social impact, psychological impact, and aesthetic concern (score range 0-92, high scores represent high negative impact)

[2] Treatment Satisfaction Questionnaire (TSQ): Customized 15 item questionnaire that measures overall satisfaction, comfort, aesthetics, speech, eating, and burden of compliance (score range 0-100).

[3] Visual Analog Scales (VAS): 100-mm pain (0=no pain), aesthetic acceptability (0=very dissatisfied), and overall satisfaction (0=very unsatisfied) scales.

## Secondary outcomes were:

[1] Oral Health Impact Profile-14 (OHIP-14): Valid OHRQoL.

[2] Clinical outcomes: Plaque index, gingival index, the incidence of white spots lesions.

Beginning with initial bonding / aligner delivery up to debonding/last aligner: Duration of treatment

## Assessment timeline:

The baseline (T0), 3 months (T1), 6 months (T2) and 12 months (T3) were assessed. A blind examiner was used to conduct clinical examinations. The questionnaires were completed in a private consultation room and thus reduced the response bias.

## Statistical analysis:

The data was analyzed with SPSS26.0. Assessment of normalcy was done through the Shapiro-Wilk test. Continuous variables of independent variables were compared using independent t-test and categorical variables were compared using chi-square test. Group-time interaction ANOVA was used to estimate temporal change. Multiple comparisons were corrected by Bonferroni. Pearson correlation was used to test the relationship between the aesthetic concern and the satisfaction. The significance level was taken to be p<0.05. The findings were in terms of Mean±SD or percentages.

## Results:

All 120 patients completed the 12-month assessment period (60 per group). Baseline demographic and clinical parameters were well- matched between groups (p>0.05). Mean age was 28.4±6.7 years in the CA group and 27.9±7.1 years in the FA group. Female representation was slightly higher in the CA group (61.7% vs 56.7%, p=0.582). PAR scores were comparable (CA: 28.4±5.2, FA: 29.1±5.8, p=0.512). Baseline PIDAQ total scores indicated similar levels of aesthetic concern (CA: 42.3±8.7, FA: 43.1±9.2, p=0.654) (Table 1). PAR: Peer Assessment Rating; PIDAQ: Psychosocial Impact of Dental Aesthetics Questionnaire; OHIP-14: Oral Health Impact Profile-14; VAS: Visual Analog Scale (0-100) PIDAQ scores demonstrated significant group differences over time (p<0.001). At 12 months, the CA group showed lower total PIDAQ scores (18.4±6.2 vs 29.7±7.8, p<0.001), indicating superior psychosocial adjustment. The aesthetic concern subscale showed the largest difference (CA: 3.2±1.4 vs FA: 6.8±2.1, p<0.001). VAS aesthetic satisfaction scores favoured CA at all-time points, with 78.3% rating aesthetics as "excellent" (VAS≥80) at 12 months versus 41.7% in the FA group (p<0.001). Repeated measures ANOVA confirmed significant timexgroup interaction (F=24.67, p<0.001) (Table 2). VAS comfort scores consistently favoured CA (p<0.001). At 12 months, CA patients reported mean comfort score of 7.8±1.4 versus 5.9±1.6 for FA. Pain severity was significantly lower in CA at T1 (3.2±1.1 vs 5.8±1.4, p<0.001) and T2 (2.1±0.9 vs 3.4±1.2, p<0.001), but comparable by T3 (1.8±0.8 vs 2.0±0.9, p=0.234). OHIP-14 scores showed greater initial deterioration in FA at 3 months (26.8±6.2 vs 21.4±5.8, p<0.001), but converged by 12 months (CA: 12.4±4.1, FA: 13.7±4.6, p=0.156). Plaque index was significantly higher in FA at 6 months (1.8±0.6 vs 1.2±0.5, p<0.001), with 31.7% of FA patients developing white spot lesions versus 11.7% in CA (p=0.008). Treatment duration was similar between groups (Table 3). Self-reported compliance was high in both groups. CA patients reported wearing aligners an average of 21.8±1.2 hours daily. Overall treatment satisfaction (TSQ total score) was significantly higher in CA at 12 months (82.4±8.7 vs 71.3±9.8, p<0.001). The social impact subscale showed the largest group difference (CA: 8.9±1.4 vs FA: 6.2±1.8, p<0.001). However, 23.3% of CA patients expressed frustration with aligner maintenance and removal during eating, compared to 8.3% of FA patients reporting dietary limitations (p=0.027).

## Discussion:

This prospective cohort study offers strong comparative figures of patient perception and satisfaction between clear aligners and fixed orthodontic appliances with unique benefits of clear aligners in aesthetical satisfaction and comfort groups and convergent results regarding the oral health-related quality of life in the long run. The implications of these findings on the treatment planning of adult orthodontics are very high. The enhanced aesthetic satisfaction described by clear aligner patients is consistent with the existing literature putting the focus on appearance as the main reason that prompts adult orthodontic treatment [2, 12]. The proportion of CA patients who rated aesthetics as excellent at 12 months (78.3 vs. 41.7) is a clinically significant difference which probably affects treatment adherence and self-esteem in the active treatment. This benefit was also maintained during treatment, unlike the results of certain studies, which hypothesize the decline of aesthetic benefits as patients get used to them [9]. The subscale scores of the PIDAQ aesthetic concern measure, and were found to remain 50% less in the CA group, support the statement that aligners significantly alleviate psychosocial distress due to conspicuous appliances [13]. Another area where the clear aligners had a clear advantage was in comfort. The prolonged increased ratings on comfort during treatment process are associated with decreased soft tissue irritation and lack of bracket/wire induced trauma [14]. The large difference in pain during the first months (T1-T2) is associated with the process of getting used to the pain of bracket irritation and the forces of the archwire, but the pressure caused by aligners is often characterized as more diffuse and bearable [15]. Nevertheless, the overlap of the pain scores at the age of 12 months indicates that the patients in both groups eventually learn the modalities that they are exposed to [16]. It is interesting to note that the constant treatment related comfort advantage of aligners failed to translate into better OHIP-14 scores at the end of treatment, which suggests that comfort is one aspect of a multifaceted quality of life measure that requires functional and psychological aspects.

The oral health impact trajectory depicted a significant trend, i.e., fixed appliance patients showed more significant initial deterioration, especially at 3 months, with OHIP-14 scores being 25 percent higher than the CA group. This early influence must be indicative of problems with speech adaptation, eating, and oral hygiene control at the time of the acclimatization stage [17]. This would be followed by the convergence after 12 months implying that patients would establish compensatory mechanisms and get used to their appliances psychologically. This time trend resembles results of a study by Li *et al.* [18], who noted that the OHRQoL impairment is the highest during the 1-3 month period in case of fixed appliances and slowly returns to normal. The increased incidence of plaque index and white spot lesion in the FA group at 6 months is evidence of the oral hygiene dilemma that has been widely documented in regards to brackets and which has potentially long term implications on the health of enamel [19]. The implication of equivalent duration of treatment between groups is that clear aligners are not necessarily slow. The probably minimal differences in efficiency were reduced by our standardized non-exclusion treatment protocols and experienced providers. Nevertheless, this observation is inconsistent with some meta-analyses that fixed appliances attain some tooth movements faster [20]. The difference could also be attributed to patient selection bias in our cohort, with more complex movements involving large root rotational forces being excluded which could be biased towards aligner efficiency. Conformity is a burning factor. Although self-reported wear time (21.8 hours) seems sufficient, the frustrations about aligner care expressed by 23.3% of CA patients demonstrate that there is a hidden cost. This compliance fatigue might get worse with extended treatments and might undermine the results with less motivated patients [21]. In contrast, fixed appliances, though subjecting patients to a diet remove the variables of wear time, which is crucial, and there will be constant force application. This core difference implies that the choice of the modality should be dictated by the specifics of personality traits and lifestyle of the patients instead of the presumed universal superiority of aligners. The results of the social impact, where CA patients reported much more improved social functioning indicate social and workplace realities of adult patients. Aligners provide a significant psychosocial benefit that goes beyond vanity even in professional situations where appearance may affect the perception of others [12]. This point holds true especially with regard to client-facing professionals and can explain the increased cost of aligner therapy in terms of quality-adjusted life year. The strengths of the study are prospective nature, standardized outcome measures, blind clinical examiner and high retention rate. The validated tools (PIDAQ, OHIP-14) increase the international literature comparability. But constraints should also be recognized. The non-randomized design creates a selection bias, in which the patients chose their treatment at their discretion and according to their financial capability. Although we balanced out baseline factors, we did not measure confounding factors like personality type and socioeconomic status, which could have affected the measurements. The 12 months of observation would capture the experiences of early to mid-treatment experiences but would fail to forecast the satisfaction patterns at the stages of retention and long-term stability. Also, the single-center and experienced provider could be a limiting factor to generalization to general dental practice. Randomized controlled designs should be used in future studies to reduce selection bias but ethical issues exist on whether these studies should be ethical in terms of patient choice in elective treatment. The long-term follow up (2-5 years) would clarify the experiences of satisfaction and retention phase. Reimbursement policies would be informed by cost-effectiveness analyses that would involve quality-adjusted life years. Also, qualitative studies that examine patient storytelling may offer a more in-depth understanding of the difference in the lived experience of modalities. Overall, the present research gives strong evidence that clear aligners are better than fixed appliances in terms of patient-reported aesthetic satisfaction and comfort in adult orthodontic patients, and converging oral health-related quality of life results are achieved after 12 months. The choice of treatment must include values, lifestyle, and complexity of malocclusion since aligners have actual psychosocial advantages but need motivated adherence.

## Conclusion:

Adults treated with clear aligners experience greater aesthetic satisfaction and comfort over 12 months, achieving similar oral health-related quality of life as those with fixed appliances. Clear aligners reduce psychosocial impact but demand higher patient compliance, whereas fixed appliances offer stable force control and suit less self-directed patients. Clinicians should engage in shared decision-making based on patient priorities and case complexity, with further long-term trials needed to refine selection criteria.

## Figures and Tables

**Table 1:** Baseline demographic and clinical characteristics

**Characteristic**	**Clear Aligners (n=60)**	**Fixed Appliances (n=60)**	**p-value**
Age (years), Mean±SD	28.4±6.7	27.9±7.1	0.701
Gender (female), n (%)	37 (61.7)	34 (56.7)	0.582
PAR score, Mean±SD	28.4±5.2	29.1±5.8	0.512
Baseline PIDAQ total, Mean±SD	42.3±8.7	43.1±9.2	0.654
Baseline OHIP-14, Mean±SD	18.6±5.4	19.2±5.8	0.543
Baseline VAS aesthetics, Mean±SD	2.8±1.2	2.6±1.1	0.398

**Table 2:** Aesthetic perception and satisfaction scores over 12 months

**Parameter**	**Group**	**Baseline**	**3 months**	**6 months**	**12 months**	p-value*
PIDAQ total	CA	42.3±8.7	28.4±7.1	22.6±6.8	18.4±6.2	<0.001
	FA	43.1±9.2	37.2±8.4	33.8±7.9	29.7±7.8	
VAS aesthetic satisfaction	CA	2.8±1.2	6.8±1.8	8.1±1.4	8.7±1.2	<0.001
	FA	2.6±1.1	4.2±1.5	5.8±1.7	6.4±1.8	
Dental self-confidence	CA	8.4±2.1	5.2±1.8	3.8±1.4	2.6±1.2	<0.001
	FA	8.7±2.3	7.1±2.0	6.2±1.9	5.4±1.7	
Values are Mean±SD (PIDAQ)
or Mean±SD (VAS 0-10);
*p-value for timexgroup interaction;
PIDAQ: Psychosocial Impact of Dental
Aesthetics Questionnaire (range 0-92,
lower scores better)

**Table 3:** Comfort, oral health impact, and clinical outcomes

**Parameter**	**Group**	**3 months**	**6 months**	**12 months**	p-value*
VAS comfort	CA	6.4±1.6	7.2±1.5	7.8±1.4	<0.001
	FA	4.2±1.4	5.1±1.6	5.9±1.6	
OHIP-14	CA	21.4±5.8	16.2±4.9	12.4±4.1	0.012
	FA	26.8±6.2	19.7±5.4	13.7±4.6	
Plaque index	CA	1.4±0.5	1.2±0.5	0.9±0.4	<0.001
	FA	1.9±0.6	1.8±0.6	1.1±0.5	
White spot lesions, n (%)	CA	3 (5.0)	5 (8.3)	7 (11.7)	0.008
	FA	8 (13.3)	14 (23.3)	19 (31.7)	
Values are Mean±SD;
*p-value for timexgroup interaction;
VAS: Visual Analog Scale (0-10, higher better);
OHIP-14: Oral Health Impact Profile-14
(range 0-56, lower better)
